# An Indole-Rich Postbiotic Reduces Itching in Dogs: A Randomized, Double-Blinded Placebo-Controlled Study

**DOI:** 10.3390/ani15142019

**Published:** 2025-07-09

**Authors:** Aylesse Sordillo, Jonna Heldrich, Raphaël Turcotte, Ravi U. Sheth

**Affiliations:** Kingdom Supercultures, Brooklyn, NY 11205, USA

**Keywords:** canine, pruritus, itching, postbiotic, microbiome

## Abstract

Many dogs suffer from pruritus, which is commonly caused by atopic dermatitis and associated with skin inflammation. The immune system and inflammatory response, and in particular the gut–skin axis, are central to the pathogenesis of atopic dermatitis. Managing atopic dermatitis involves complex, iterative treatment plans; early strategies supporting gut–skin health are needed to prevent elevated itching from progressing toward a disease condition requiring drug therapy. This double-blind, placebo-controlled, randomized trial evaluated the ability of a novel, indole-rich canine immune health postbiotic (CIHP) to reduce itching and promote a healthy gut microbiome in dogs with subclinical, but elevated itching behavior. Thirty dogs were stratified into two groups based on baseline scratching frequency, receiving either CIHP or placebo as a powder topper for 28 days. Canine itching was evaluated through accelerometer-based tracking and the Pruritus Visual Analog Scale (PVAS) score on Days 0, 7, 14, 21, and 28. Skin and coat health was assessed on Days 0, 14, and 28, and the gut microbiome was sequenced from fecal samples on Days 0 and 28. CIHP reduced scratching by 20% relative to the baseline (*p* = 0.032) and PVAS score by 27% compared to the placebo (*p* = 0.02). CIHP improved skin and coat quality compared to the placebo at Day 14 (*p* = 0.01) and increased Shannon diversity by 4.6% (*p* = 0.043), shifting gut microbiome composition. These findings validate this postbiotic’s ability to reduce itching in dogs with subclinical, but elevated itching behavior, provide evidence of promoting a healthy gut–skin axis, and suggest potential as an early intervention in the context of pruritic conditions, as well as for broader immune-related benefits.

## Simple Summary

Canine itching is a common condition most often caused by atopic dermatitis, a chronic skin condition. The pathogenesis of atopic dermatitis involves immune and inflammatory responses, with the gut–skin axis playing a crucial role. Current atopic dermatitis treatment is typically complex and iterative. Nutritional or supplement interventions supporting gut–skin health could help to reduce abnormal itching in dogs before it becomes pathological, and thus potentially reduce or delay the need for medical interventions. Postbiotics including indole compounds, a class of well-characterized beneficial microbial metabolites, offer a promising approach to influence the gut–skin axis. A double-blind, randomized clinical trial evaluated an indole-rich postbiotic in dogs with subclinical, but elevated itching behavior. Results showed a 20% reduction in scratching relative to the baseline and a 27% decrease in human-perceived itching (Pruritus Visual Analog Scale) compared to the placebo at Day 28. The intervention also improved skin and coat quality at Day 14 and increased gut microbiome diversity at Day 28. These findings indicate that this indole-rich postbiotic supports a healthy gut–skin axis, and it suggests potential broader applications for support of immune-related conditions.

## 1. Introduction

Pruritus, or itching, is a common condition in dogs that is most often caused by atopic dermatitis, a complex, hereditary, and chronic skin disease that affects a large number of dogs [1,2,3,4]. In addition to pruritus, atopic dermatitis is characterized by inflammation and a hypersensitivity to environmental antigens [2,5]. Atopic dermatitis is multifaceted, with different subgroups and phenotypes, and is influenced by genetic and environmental factors [6,7,8,9,10].

The immune system and inflammatory response play crucial roles in the pathogenesis of atopic dermatitis [11,12,13]. In particular, the gut–skin axis influences its pathogenesis, as gut dysfunction and alterations to the gut microbiome can influence both immune responses and skin barrier function [14,15]. Comparisons of the gut microbiome of healthy and atopic dogs have found that atopic dogs have lower gut microbiome diversity and gut microbiome dysbiosis, indicating that alterations in the gut microbiota diversity and composition may be associated with atopic dermatitis [16,17,18]. In addition, a decrease in gut barrier integrity has been shown to increase antigen transfer, altering the immune response [19,20,21]. Studies in children with eczema and atopic dermatitis found them to have higher intestinal permeability than their healthy counterparts, and increased intestinal permeability is a risk factor for food allergy in dogs, of which the main complaint is itching [20,21,22]. Moreover, another study on atopic dogs found that serum levels of intestinal alkaline phosphatase and trefoil factor 3, which indicate intestinal damage and inflammation in dogs, were significantly increased compared to healthy controls, suggesting that the atopic dogs suffered from intestinal damage [23]. In line with these findings, both small intestinal bacterial overgrowth and irritable bowel syndrome are associated with skin inflammation in humans [24,25]. Together, these findings indicate that supporting a healthy gut microbiome in itching dogs may help attenuate the pathogenesis of atopic dermatitis.

Microbially-derived ingredients show great potential to mitigate factors associated with the pathogenesis of atopic dermatitis, such as itching, by supporting a healthy gut–skin axis and broader immune system, and thus, they may be good candidates to impact early symptoms associated with the development of the condition [26,27,28]. In humans, probiotics have been shown to both improve atopic dermatitis symptoms and modulate the gut microbiome, and there is also some evidence that they may be effective in dogs [26,27,28]. In dogs diagnosed with atopic dermatitis, *Lactobacillus sakei* probiotic (proBio65, Probionic) decreased the Pruritus Visual Analog Scale (PVAS) score by 10% compared to the placebo group at Day 30, but not Day 60, and reduced the Canine Atopic Dermatitis Extent and Severity Index (CADESI-03) score over two times more than the placebo at Day 60 [26]. Additionally, a dietary supplement containing a blend of probiotics, prebiotics, vitamins, nutrients, and a *Saccharomyces cerevisiae* postbiotic (EpiCor^®^, Cargill Inc.) decreased the digital PVAS score by 10% compared to the placebo at Day 14 in client-owned dogs with pruritic dermatitis, but not at other timepoints (Days 28, 42, 56, 70) [28]. It also supported a healthy gut microbiome by increasing the abundance of three probiotic taxa and reducing the abundance of taxa associated with negative effects at Day 70 [28]. However, the live cultures in probiotics are vulnerable to the temperature, pressure, and moisture conditions during pet food manufacturing and storage, as well as the varying pH levels and enzymes found throughout the digestive system [29,30,31]. These stability issues limit their overall inclusion in pet food products, treats, and supplements.

Postbiotics are a promising microbially-derived alternative for supporting a healthy gut–skin axis. A postbiotic is an inanimate probiotic, and its metabolite constituents that confer a health benefit on the host as well as some of their constituents are more stable than probiotics, facilitating incorporation into a range of products [32]. Additionally, postbiotics deliver beneficial compounds directly to the gut, limiting the potential for diet and microbiome, which vary amongst individual dogs, to influence efficacy. Nevertheless, there is very limited clinical evidence of their effectiveness at reducing itching in dogs [28,33,34]. A beta-glucan rich *Saccharomyces* postbiotic (MacroGuard^®^, Biorigin) failed to improve pruritus in client-owned dogs with signs of atopic dermatitis at Day 56, according to a scale-based observational survey [34]. In a similar study, an *Enterococcus faecalis* postbiotic did not improve PVAS score from the baseline or compared to the placebo group in client-owned dogs diagnosed with atopic dermatitis at Day 84 [33]. While intervention with a *Saccharomyces cerevisiae* postbiotic (EpiCor^®^, Cargill Inc.) in combination with a blend of probiotics, prebiotics, vitamins, and nutrients decreased the digital PVAS score by 10% compared to the placebo at Day 14, it failed to show improvement at later timepoints, and it is unclear if the postbiotic itself drove the observed effect [28]. Additionally, it failed to significantly drive an increase in gut microbiome diversity (as indicated by the Shannon diversity metric) at Day 70 [28].

Indoles are a class of metabolites produced by healthy gut microbiota through tryptophan metabolism and circulate throughout the body to deliver key systemic immune and skin health benefits [35,36]. They are well-characterized to activate the aryl hydrocarbon receptor (AhR), an important master regulator of immune and inflammatory responses, which has been linked to itch control [37,38,39,40,41]. Indole activation of AhR is known to regulate host-microbe interactions and immune function [42,43,44], while research has demonstrated that AhR activation through indoles shows significant therapeutic promise for inflammatory skin conditions, including psoriasis and atopic dermatitis [45,46]. The successful development of AhR-targeting medications like tapinarof further validates AhR as an important therapeutic target for inflammatory skin disorders—tapinarof has been shown to reduce itching in humans [45,47]. Considering (1) the link between indoles and AhR activation and (2) the link between AhR activation and itching in mammals, we hypothesized that indoles may be capable of supporting a healthy gut–skin axis to reduce itching in mammals, including dogs.

In this work, we evaluated the ability of a novel, indole-rich canine immune health postbiotic (CIHP) to support a healthy gut–skin axis and reduce scratching in dogs. More specifically, we performed a double-blind, placebo-controlled, randomized clinical trial in a dog population displaying elevated itching behavior, but with no dermatologic or systemic condition diagnosis, in order to assess the potential of CIHP in the context of early intervention to pruritus.

## 2. Materials and Methods

### 2.1. Animals

The study was conducted at a registered research facility that complied with all local regulations governing the care and use of laboratory animals and was conducted in accordance with OMAFRA, the CCAC Guide to the Care and Use of Experimental Animals. To ensure compliance, the protocol was reviewed and approved by the facility’s Institutional Animal Care and Use Committee (IACUC).

All dogs were part of a permanent colony made up of beagles and small mixed breed dogs. All participants were between 1 and 7 years old. Both intact and neutered animals of both sexes were included in the study. Dogs were allowed to socialize in groups in outside dog runs for at least one hour each day and had access to a larger dog park at least twice weekly for robust play and exercise.

All dogs were pair-housed in 10-foot × 10-foot runs that could be divided into 5-foot × 10-foot runs for individual housing. Beds and blankets were provided to all dogs. Fresh, clean, drinking water was provided ad libitum. Dogs remained in the same room for the duration of the study. Animal rooms were cleaned at least once daily, disinfected twice weekly, and descaled when needed.

All dogs were classified as USDA Category C for the full duration of the study: Animal use activities that involve no more than momentary or slight pain or distress for which there is no need for use of pain-relieving drugs.

### 2.2. Study Design

The study was a double-blind, placebo-controlled, randomized trial. No itching treatment, including supplements, products, or medical baths to relieve itching, allergies, or atopic dermatitis were administered for the duration of the study. The duration of the study was 48 days (Day −19 to 28, Figure S1), and the intervention was administered for 28 days (Day 1 to 28). The study employed a multi-phase screening process in which dogs were first enrolled based on initial eligibility criteria (enrollment), followed by further narrowing of the population based on (1) data quality during the observation period and (2) itching behavior during the baseline period.

Dogs enrolled in this study were part of a dog population with subclinical, but elevated itching behavior. This population is defined by general and skin-specific health eligibility criteria. The generic health eligibility criteria were as follows: the dog (1) is within the healthy weight range for its breed, and (2) is not taking any prescribed medications beyond standard preventative flea, tick, and heartworm medication. The skin-specific health eligibility criteria were the following: the dog (3) is not currently diagnosed with a chronic yeast infection of the skin, (4) is not currently diagnosed with mange, (5) does not currently have fleas or other parasitic infections, (6) has not been prescribed medication for any allergy or atopic dermatitis by a veterinarian in the past year, and (7) does not currently take any supplements or products to relieve itching, allergies, or atopic dermatitis. With respect to the itching behavior, the eligibility criterion was as follows: (8) the dog’s itching is perceived as elevated by the technicians at the research facility—this itching behavior was naturally occurring (i.e., not experimentally induced). Taken together, the last three criteria imply that itching was elevated but insufficient to be categorized as a clinical health issue. An enrollment criteria around age was also used: (9) the dog is over 1 year and below 13 years old.

The first further narrowing of participants was based on a quantitative assessment of itching behavior during the observation period (Day −19 to −13). This assessment was performed by tracking scratching frequency with an accelerometer device (Whistle™ Health 2.0 Smart Device, Mars Petcare, Franklin, TN, USA). This accelerometer device was validated to accurately detect canine behaviors, including itching behaviors such as scratching [48]. The inclusion criteria for this first narrowing of participants was producing high accelerometer device data quality (≥50% of the data is recorded, as opposed to imputed) during the observation period.

The second further narrowing of participants was based on a quantitative assessment of itching behavior during the baseline period (Day −6 to 0). In order to focus itching behavior analysis on a healthy dog population with noticeable itching, a dog was included in this analysis if its scratching was between 53–299 s/day based on the recorded weekly average scratching during the baseline period. This interval is based on the manufacturer’s analysis of a consumer dog population of ten thousand dogs and corresponds to 94% of itchy dogs, respectively, and 47% of all dogs, respectively [48]. This second narrowing was implemented to exclude dogs displaying potentially clinical levels of itching behavior, and to focus on dogs displaying levels of itching that do not indicate a potential need for a veterinary intervention.

Thirty-six dogs were enrolled and initially outfitted with the accelerometer device. Thirty dogs were included in the intervention cohort and stratified randomly into 2 groups of 15 subjects based on the retrospective weekly average of scratching seconds per day from Day −19 to Day −13 and sex. Of these thirty dogs, a subset was included in the analysis of itching behavior (Table 1). All participants were bathed and shampooed (Aloe and Oatmeal Shampoo, ProCleanse, Guelph, ON, Canada) once during the study, after the stratification and prior to intervention.

There were two termination criteria for the study: (1) Abnormal changes in a dog’s health as assessed by a veterinarian followed by a recommendation by a veterinarian to remove the dog from the study. (2) Refusal to eat more than 4 consecutive meals containing the ingredient (not eating over the course of 2 days).

### 2.3. Intervention

CIHP is a commercially available ingredient (Superculture^®^ Pet Immune ingredient, Kingdom Supercultures, Brooklyn, NY, USA) composed of a tapioca maltodextrin carrier and dried *Pediococcus acidilactici* fermentation product. The fermentation product is heat treated to inactivate live cells, then spray or freeze dried. The placebo was tapioca maltodextrin (Mike’s Mix), the same carrier utilized in the ingredient.

On Days 1–28, each dog received a 75-mg dose of CIHP or the placebo added to both their first and second meal of the day (standard dry diet; Purina Dog Chow) as a powder topper, for a total daily dose of 150 mg. The daily food portion was placed into a bowl and sprayed with enough water to moisten the food and promote adhesion of the powder topper to the food. The dog was served the food containing the powder topper and given up to 30 min to eat all the food. The study employed a double-blind design, in which the individuals performing intervention administration, data collection, or data analysis were blind to group assignments.

### 2.4. Scratching Frequency Quantification via Accelerometer Device

All dogs were continuously outfitted with an accelerometer device for the duration of the study. The devices were regularly synced to the accelerometer device application using smart phones (Samsung Galaxy A03, Suwon-si, South Korea) and were charged as needed. Scratching frequency data was subsequently downloaded from the accelerometer device application programming interface (API).

The data were processed according to manufacturer recommendations and best practices utilized in their marketed consumer application. Statistical analysis was performed on the retrospective weekly average. The quality control filter for each day of data is that ≥50% of the data is recorded (as opposed to imputed).

To account for occasional days of lost or low-quality data that did not pass the quality control filter, a minimum window of 5 days was used. All participants included in the statistical analysis had 5–7 days of high-quality data for each of the time bins listed, inclusive: (1) Days −6–0, (2) Days 1–7, (3) Days 8–14, (4) Days 15–21, and (5) Days 22–28.

Based on the pre-registered study preference for study inclusion, severe scratchers (dogs that scratched on average at least 300 s per day during Days −6–0; 30% of participants) were removed from the analysis, and dogs with subclinical scratching (dogs that scratched 53–299 s/day) were included in the analysis (see Section 2.2).

The following statistical analyses were performed on the scratching frequency data: A Shapiro–Wilke test was used to assess if the data were normally distributed. A two-tailed paired *t*-test or Wilcoxon test was performed on the average of each participant to assess how scratching frequency changed from the baseline within a group. The absolute and relative changes from the baseline were calculated for each participant on Day 7 and Day 14. A two-tailed unpaired *t*-test or Mann–Whitney test on the absolute and relative changes in scratching frequency were used to assess any differences between the two groups.

### 2.5. Pruritus Visual Analog Scale Scoring

On Days 0, 14, and 28 a single technician filled out a Pruritus Visual Analog Scale (PVAS) for each dog by marking the continuous scale (Figure S2) [49,50]. The scoring was carried out based on an observation log posted outside of each room where the technician could make notes/observations throughout the day. The log was signed off on a minimum of twice daily, but the technicians observed the dogs during all daily activities (morning check, during feeding, while outside, afternoon check, etc.). The technician did not have access to the accelerometer device data. Each dog’s score was then recorded in a Microsoft Excel sheet.

The following statistical analyses were performed on the PVAS data: A Shapiro–Wilke test was used to assess if the data were normally distributed. A two-tailed paired *t*-test or Wilcoxon test was performed on the average of each participant to assess how the PVAS score changed from the baseline within a group. The absolute and relative changes from baseline were calculated for each participant on Day 7 and Day 14. A two-tailed unpaired *t*-test or Mann–Whitney test on the absolute and relative change in PVAS score was used to assess any differences between the two groups.

### 2.6. Skin and Coat Quality Assessment

On Days 0, 14, and 28 a single technician filled out the Skin and Coat Survey (Figure S3) for each dog, which is a standard survey [51,52,53]. The technician did not have access to the accelerometer device data.

The following statistical analysis was performed on the skin and coat quality assessment data: the data at Day 7 and Day 14 were compared to the Day 0 data and classified in a three-way contingency table as “improved”, “worsened”, or “unchanged”. A Fisher’s exact test was performed on the contingency table data.

### 2.7. Fecal Sampling

On Days 0 and 28 a fecal sample was collected from the first evacuated feces representing the first defecation of the day as follows: Wearing clean gloves, the collection tube (Zymo Research DNA/RNA Shield Fecal Collection Tube Cat. #R1137, Irvine, CA, USA) was labeled with the subject identifier and date and time of sampling. The tube was opened, being careful not to touch the scoop attached to the cap. One level scoop of feces was collected into the tube, and the cap was screwed tightly to recap. The tube was shaken vigorously until the sample was completely mixed. Samples were stored at room temperature until sequencing per manufacturer recommendations.

### 2.8. Gut Microbiome Sequencing

Fecal samples were processed altogether in a randomized order in a multi-well plate. Samples were processed in duplicate from the raw fecal samples. The V4 hypervariable region of the 16S rRNA gene was amplified with barcoded sequencing primers and equal volumes of the resulting libraries were pooled and sequenced on the Illumina iSeq (Illumina, San Diego, CA, USA), using iSeq Control Software (Version 3.1.0.1952). Counts of the zero-radius operational taxonomic units (ZOTUs) [54] were determined for each library using USEARCH (https://www.drive5.com/usearch/download.html (accessed on 24 August 2024)), and taxonomic assignments for the ZOTUs were made using the RDP classifier [55]. Sequences of taxonomic assignments with low confidence (<70% confidence) were searched on NCBI BLAST (Version 2.16.0) (https://blast.ncbi.nlm.nih.gov/Blast.cgi?PROGRAM=blastn&PAGE_TYPE=BlastSearch&LINK_LOC=blasthome (accessed on 9 December 2024)) and BLAST taxonomy (Table S1). Raw read counts were used to calculate alpha diversity metrics. For fold change calculations, taxa with less than 10 reads in at least 20% of samples were removed, and read counts were standardized to the median sequencing depth. Read counts were analyzed in R using phyloseq (Version 1.46.0) [56] and DESeq2 (Version 1.42.1) [57] packages.

Alpha diversity describes the variety of individuals that make up a community on a local scale [58]. In this study, alpha diversity was assessed by three measures: Observed (richness), Evenness (Pielou), and Shannon diversity. Observed diversity is a simple count of the number of taxa, or richness of taxa, found in the community. Evenness describes the relative abundance of the taxa found in the community. The Shannon diversity index is a measure that accounts for both the evenness and richness across the community. A paired two-sided Wilcoxon test was used to compare changes in alpha diversity metrics over time.

The log fold change in number of reads was calculated for each taxa for each group of dogs. Taxa are considered significantly changed if the absolute value of the log fold change from Day 0 to Day 28 is greater than 0.55 with a False Discovery Rate (FDR)-adjusted *p*-value less than 0.001.

## 3. Results

No adverse events were recorded, and no participants were removed from the study.

**Figure 1:**
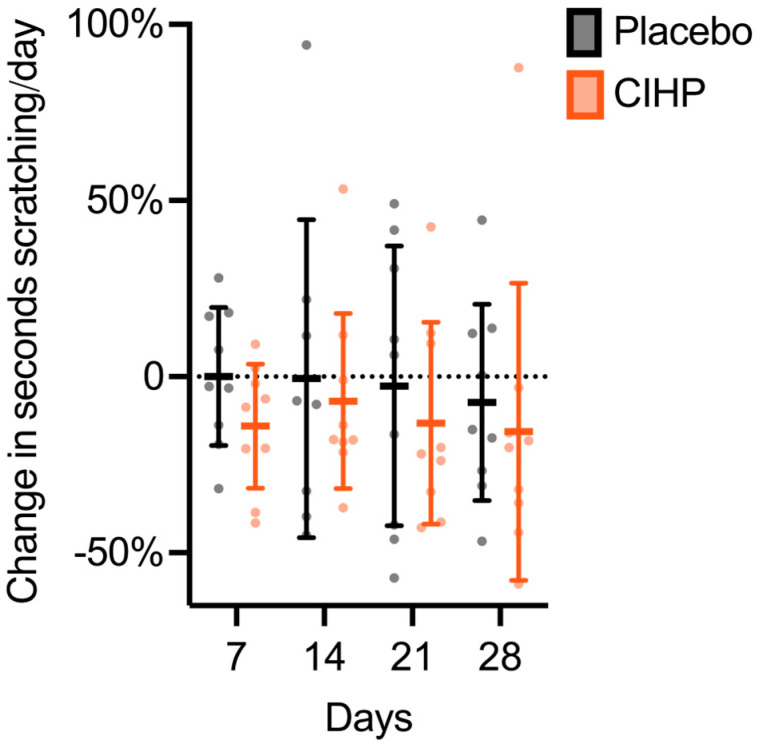
CIHP reduced seconds scratching per day at Day 28 relative to baseline. Lines and error bars indicate the mean and standard deviation. Points represent individual participant data.

CIHP effectively reduced scratching in dogs with subclinical, but elevated itching behavior (dogs that scratched 53–299 s/day during the observation period, see Section 2.2) within the duration of the study as assessed by the accelerometer device (Table 1 and Figure S4). CIHP reduced scratching by 20% ± 24% relative to baseline (Figure 1, *p*-value = 0.032, two-tailed paired *t*-test). There was no change in scratching in the placebo group (*p*-value = 0.82, two-tailed paired *t*-test).

**Figure 2:**
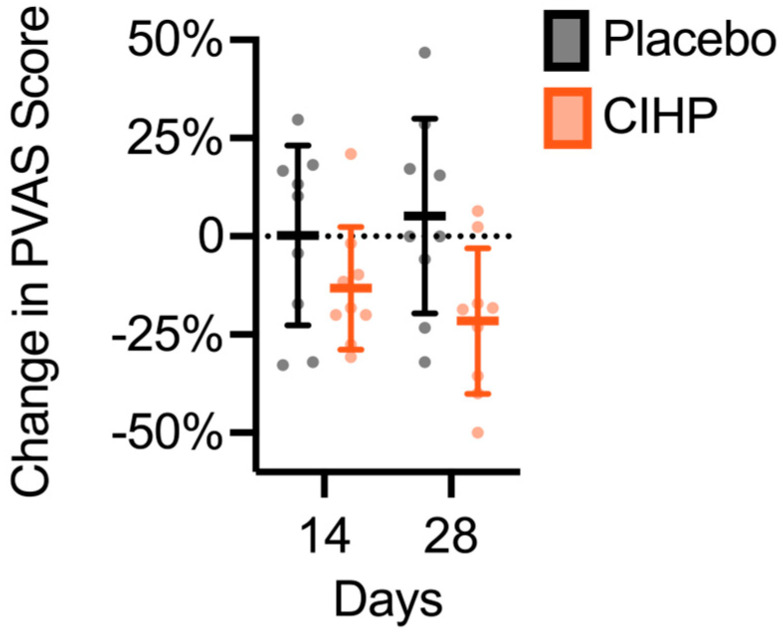
CIHP reduced PVAS Score at Days 14 and 28. Lines and error bars indicate the mean and standard deviation. Points represent individual participant data.

In the same population of dogs with subclinical, but elevated itching behavior, CIHP reduced the score, relative to the baseline, by 14% after 14 days of intervention and by 17% after 28 days of intervention (Figure 2, *p*-value = 0.03 and *p*-value = 0.0095, two-tailed paired *t*-test). There was no change in the PVAS score in the placebo group (*p*-value = 0.73, the two-tailed paired Wilcoxon test, and *p*-value = 0.45, the two-tailed paired *t*-test). At Day 28, the PVAS score change from baseline was significantly lower in the CIHP group compared to the placebo (mean difference = 27%, *p* = 0.02, two-tailed unpaired *t*-test).

**Figure 3:**
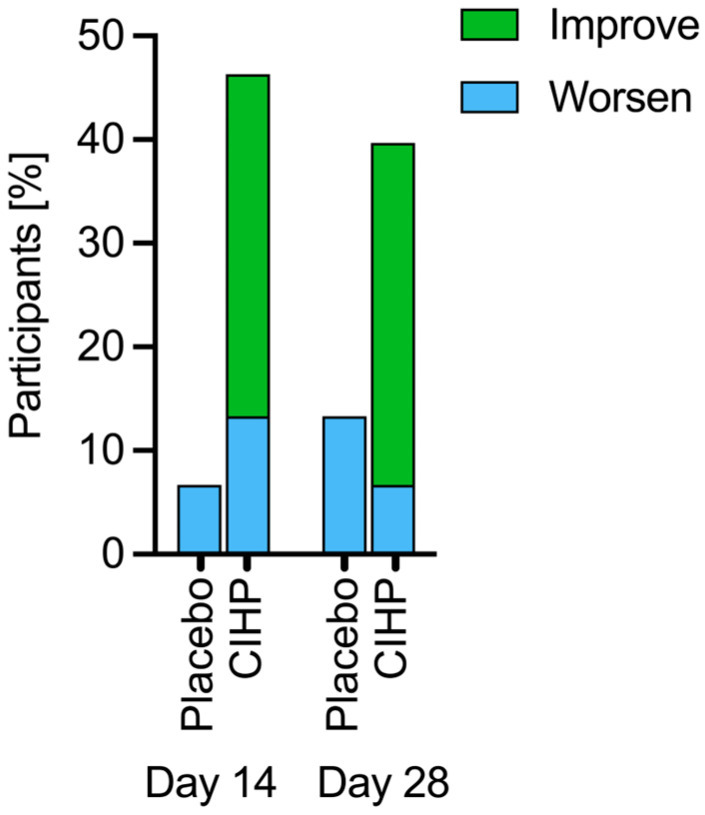
CIHP improved human perception of coat quality at Days 14 and 28. Both groups started with a median score of 1 (mean = 1.33), indicating a typical quality coat. Participants (33%) had an improved coat quality relative to baseline at Days 14 and 18; none of the participants in the placebo group improved.

In addition, CIHP was effective in improving coat quality as shown by more improvements in the scores compared to the placebo at Day 14 (Figure 3, *p*-value = 0.01, mean difference = 3%, Fisher’s exact test) and at Day 28 (*p*-value = 0.02, mean difference = 11%, Fisher’s exact test).

**Figure 4:**
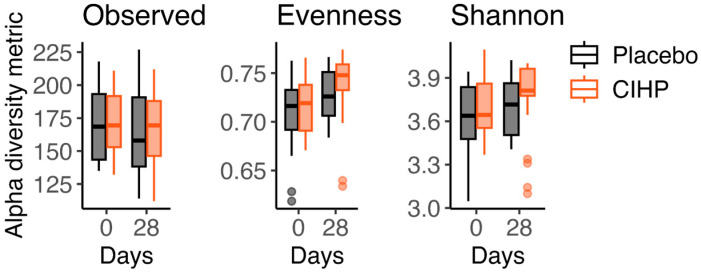
Alpha diversity metrics increase with CIHP intervention. Evenness and Shannon diversity increased by a higher magnitude with CIHP intervention compared to placebo. Observed diversity did not change for either group.

CIHP also increased Shannon diversity by 4.6% at Day 28 (*p*-value = 0.043, two-tailed paired Wilcoxon test; Figure 4, Table 2). There was no change in Shannon diversity of the placebo group (*p*-value = 0.092). While the observed diversity and evenness followed similar dynamics for the two groups throughout the study, with the observed diversity remaining the same and evenness increasing significantly (Figure 4, Table 2), the magnitude and level of significance of the increase in evenness were greater for CIHP than for the placebo group (CIHP: 4%, *p*-value = 0.00095; Placebo: 1.4%, *p*-value = 0.015; two-tailed paired Wilcoxon test).

**Figure 5:**
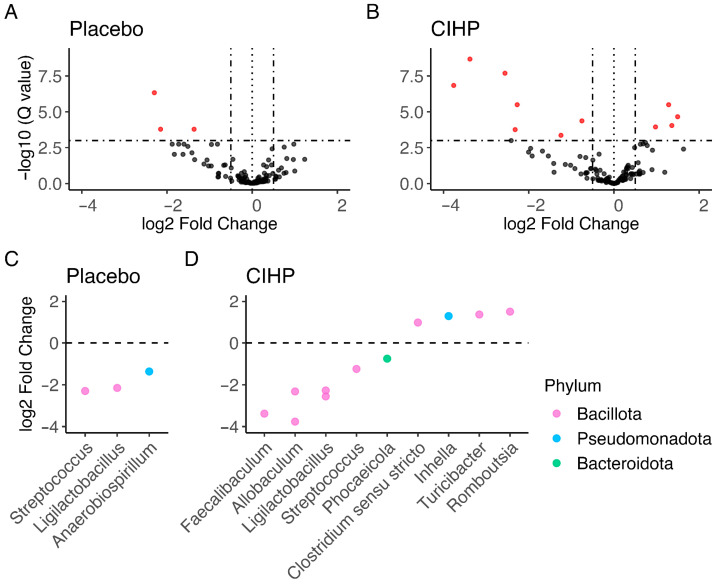
Fold changes in the microbiome over the course of the study. The volcano plots show more significant changes in the abundance of taxa (red dots) occurring with (**B**) CIHP intervention than with (**A**) the placebo over the course of the 28-day study. The horizontal dash–dot lines indicate FDR corrected *p*-value cutoff of 0.001 for log fold changes greater than +/−0.55, indicated by the vertical dash–dot lines. More taxa change significantly over the course of the study for (**D**) the CIHP intervention group than for (**C**) the placebo group (FDR corrected *p*-value less than 0.001 for log fold change from day 0 to day 28 is plotted).

Additionally, more taxa increased and decreased in abundance over the course of the study for dogs in the CIHP group than in the placebo group (Figure 5A,B). Three taxa significantly decreased in abundance in the placebo group, while about four times more taxa exhibited changed abundance in the CIHP group (alpha = 0.01; 7 taxa decrease and 4 taxa increase). Of the three taxa that decreased in abundance with the placebo, two are shared with the CIHP group (*Streptococcus* and *Ligilactobacillus*) (Figure 5C,D). Four taxa increased in abundance in the CIHP group, including the following genera: *Clostridium*, *Romboutsia*, and *Turicibacter*. No taxa increased in the placebo group. Overall, the shift in the gut microbiome was more substantial for dogs treated with CIHP than with the placebo.

## 4. Discussion

Itching is a common condition in dogs that is often caused by atopic dermatitis, a chronic and highly prevalent inflammatory disorder in dogs that decreases quality of life and may disrupt the relationship between dogs and their owners [1,2,59,60,61,62,63,64]. The current approach to treatment is often complex, prolonged, and individually tailored, and in severe cases, involves immunomodulatory drugs, which create barriers to adoption [11,13,51,65,66,67]. As gut health influences both the inflammatory response and skin barrier function via the gut–skin axis, microbially derived ingredients are well-suited to address canine atopic dermatitis and itching [14,15,68]. In particular, indoles are microbial metabolites that are well-suited to modulate the gut–skin axis, as they are known to be produced by a healthy gut microbiome and circulate throughout the body to deliver key systemic immune and skin health benefits by promoting a healthy inflammatory response [35,36]. The purpose of this study was to investigate the ability of a novel, indole-rich postbiotic ingredient to support a healthy gut–skin axis and to reduce scratching in dogs displaying elevated, but subclinical itching behavior as a potential early intervention to pruritic conditions.

CIHP reduced itching behavior by 20%, as quantified by a wearable accelerometer device, and reduced human perception of itching behavior in just 14 days, resulting in a change in PVAS score that was 27% lower than the placebo at Day 28. PVAS is the gold-standard survey for characterizing itching in dogs [49,50,69,70]. To the best of our knowledge, these results suggest that CIHP outperforms other pet biotics by over 2.5 times on itching reduction; comparing our results to other placebo-controlled studies on biotic ingredients for itching, five studies on either probiotics or postbiotics showed no significant impact on itching, and two probiotics showed only a 10% reduction in PVAS score after 14 or 28 days of intervention [26,28,33,34,71,72,73]. Other meta-analyses of food and supplement ingredients echo these findings and highlight a lack of strong evidence on the efficacy of existing solutions [74]. The effectiveness of CIHP provides valuable insights into the ability of targeted postbiotics to ameliorate canine itching.

In addition to reducing human perception of itching, CIHP was also found to improve human perception of overall skin and coat quality at Day 14 and Day 28, suggesting that CIHP noticeably improved the skin and coat. Improvement to skin quality may be due to reduction in itching, which can reduce skin lesions and secondary infections due to scratching [75,76]. In addition, skin quality improvement could be the result of direct improvement of the skin barrier or the support of a healthy inflammatory response [14,68]. Improvement in skin hydration and barrier function in dogs, as indicated by analytical measurements, has been shown to be associated with an improvement in human perception of skin and coat quality, albeit using a different survey than the one used in this study [77]. While a *Lactobacillus sakei* probiotic (proBio65, Probionic) reduced the CADESI-03 score—this assesses the severity of skin lesions based on erythema, excoriations, lichenification, and alopecia—over two times more than placebo at Day 60, no other probiotics or postbiotics have demonstrated improvement in overall skin and coat quality, and none have demonstrated improvement within 14 days [8,26,27,28,33,34].

Increased microbial diversity is generally understood to be associated with microbiome robustness, resilience, and health [78]. Additionally, Shannon diversity has been found to be higher for healthy dogs than for dogs with either inflammatory bowel disease [79] or atopic dermatitis [16,17]. A study using comparable analysis to ours reported about a 7% increase in Shannon diversity in healthy control dogs compared to those with atopic dermatitis, and another study indicated that oclacitinib treatment in dogs with atopic dermatitis shifted gut microbiome composition toward that of healthy controls [16,17]. While probiotic intervention in dogs has shown an increase in microbiome diversity as determined by observed taxonomic units, other studies treating dogs with postbiotics have failed to observe a change in Shannon diversity [80,81,82]. Taken together with our result that CIHP reduced itching, the evidence suggests that indole-rich postbiotics like CIHP are able to modulate the gut–skin axis by promoting a healthy gut microbiome and may be more efficacious than other microbially-derived solutions.

We also observed that CIHP increased the abundance of taxa in the gut microbiome compared to no increases in the placebo at Day 28. Many of the taxa that shifted in abundance over the course of the study are core or common members of the canine gut microbiome [83,84]. However, the taxa that increased in abundance in the CIHP group also include taxa associated with indoles—*Clostridium* and *Romboutsia* [85,86]—and the indole compounds these taxa are associated with are able to activate AhR [85,87,88,89]. Additionally, *Clostridium* supports gut health by producing indole propionic acid, which is anti-inflammatory and increases intestinal barrier integrity [90,91,92]. Consistent with our study, using an indole-rich postbiotic, studies in mice have shown that not only oral administration of an indole-producing probiotic modulated gut microbial composition and elevated tryptophan metabolism, but also that direct oral administration of indoles increased the abundance of tryptophan-metabolizing bacteria and increased indole production through microbial cross-feeding [85,86]. These results indicate that CIHP not only supports a healthy gut microbiome, but suggest that it may increase production of beneficial indoles in the gut.

The focus of this study was to characterize the impact of CIHP on canine itching, which we evaluated through two distinct types of measurement: accelerometer device behavioral tracking and observational survey scoring by a technician. While the study was conducted with animals in a research colony, the study population falls within itching levels comparable to those found in the broader dog population (94% of itchy dogs) [48]. The study was conducted using dogs with elevated itching that were otherwise healthy, and the itching across participants was likely triggered by heterogeneous internal and external factors, such as exposure to different allergens or disruptions to the gut or skin microbiome. Future studies could confirm this effect in distinct populations of dogs or evaluate if higher doses of CIHP can impact the appearance of skin in dogs diagnosed with atopic dermatitis or other dermatologic conditions. A longer observational period or a larger initial cohort may have allowed us to include a larger target population in our analysis. Additionally, using a larger cohort would have allowed us to further stratify by additional variables, such as baseline PVAS score. With regards to the PVAS assessment, it should be noted that it was developed for assessment by pet owners based on continuous observations [59,60]. In this study, the PVAS score was assessed by a technician who potentially observed dogs less frequently than would a pet owner. However, this approach has the benefit of there being a single trained observer, which mitigates the issues of reproducibility and reliability associated with this type of visual behavioral assessment [93]. Also, only the relative change in PVAS data was reported as the absolute values may not be comparable to values obtained in at-home studies. While coprophagy was closely monitored and only one event was recorded for a dog that was not paired with another study participant, avoiding co-housing would have been preferred as coprophagy may introduce confounding effects on the fecal microbiome. Another limitation of this study is that we did not assess skin lesions or take subjective measures of skin barrier function, and we did not evaluate other markers of gut and immune health, such as inflammatory cytokines.

## 5. Conclusions

This clinical study demonstrated CIHP’s ability to support a healthy gut–skin axis and reduce scratching in dogs with subclinical, but elevated itching behavior. CIHP reduced scratching by 20%, as quantified by a wearable accelerometer device and reduced human perception of itching behavior in just 14 days, resulting in a change in the PVAS score that was 27% lower than the placebo at Day 28. In addition to reducing itching, CIHP also improved the perception of coat quality at Day 14. CIHP also supported a healthy gut microbiome, increasing Shannon diversity at Day 28 and increasing taxa that support skin, gut, and immune health. Together, the results indicate that CIHP, an indole-rich postbiotic, supports a healthy gut–skin axis and suggest it may have broader applications in nutritional support for immune-related conditions.

## Figures and Tables

**Table 1:** Daily scratching frequency (mean ± std) throughout the study.

		Timepoint				
	Group	Day 0	Day 7	Day 14	Day 21	Day 28
Scratching [s/day]	CIHP	151 ± 78	144 ± 92	136 ± 80	132 ± 80	128 ± 77
	Placebo	184 ± 52	185 ± 70	196 ± 140	192 ± 116	180 ± 96
Sex ratio [female:male]	CIHP	2:8	2:7	2:8	2:7	2:7
	Placebo	4:5	4:5	3:5	4:5	4:5
Participants * [n]	CIHP	10	9	10	9	9
	Placebo	9	9	8	9	9

* All participants with elevated itching that passed data QC.

**Table 2:** Changes in diversity metrics from Day 0 to Day 28 of the study.

Group	Measure	Observed	Evenness	Shannon
Placebo	*p*-value	0.12	0.015	0.092
	% change	−6.2	1.4	2.1
CIHP	*p*-value	0.21	0.00095	0.043
	% change	0.0	4.0	4.6

## Data Availability

Datasets are available upon reasonable request from the authors.

## Supplementary Materials

The following supporting information can be downloaded at: https://www.mdpi.com/article/10.3390/ani15142019/s1, Figure S1: Study Timeline, Figure S2: PVAS Survey, Figure S3, Skin and Coat Survey Scores and Descriptions, Figure S4: Daily scratching frequency throughout the study, and Table S1: Taxa identities (taxa with low identity confidence).
