# Supplementary material for: An Indole-Rich Postbiotic Reduces Itching in Dogs: A Randomized, Double-Blinded Placebo-Controlled Study
Source: Animals (Basel). 2025 Jul 9;15(14):2019. doi: 10.3390/ani15142019 (PMC12291873; doi:10.3390/ani15142019)
Supplement: Supplementary file 1 [file animals-15-02019-s001.zip › animals-3696489-supplementary.pdf]

**Supplemental File S1.**

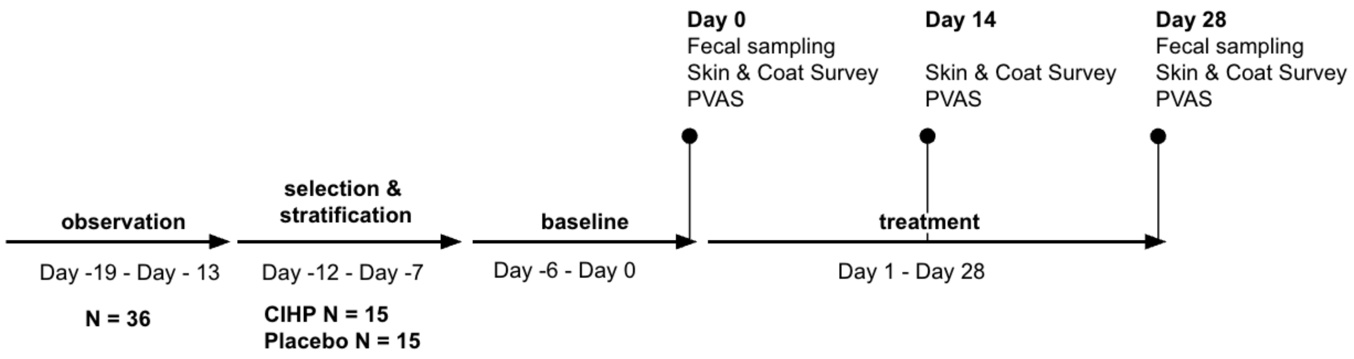


**Figure S1. Study Timeline**

10 **Extremely severe almost constant itching.** Itching is frequently observed in all situations and doesn't stop whatever is happening. The dog needs to be restrained from itching.

9

8 **Extremely severe almost constant itching.** Itching is frequently observed in all situations and doesn't stop whatever is happening. The dog needs to be restrained from itching.

7

6 **Moderate itching/regular episodes.** Itching is observed throughout the day/night. The dog doesn't itch when sleeping, eating, playing, exercising, or being distracted.

5

4 **Mild itching/ a bit more frequent**. Itching is sometimes observed at night. The dog doesn't itch when sleeping, eating, playing, exercising, or being distracted.

3

2 **Very mild itching/ only occasional episodes.** The dog is slightly itchy.

1

0 **Normal dog.** I don't think there is a problem.

**Figure S2. PVAS Survey.** The scale was accompanied by the following prompt: This scale is designed to measure the severity of the dog's itching. Itching can include scratching, biting, licking, chewing, nibbling or rubbing. Please read all the descriptions below carefully, starting at the bottom. Then use a marker to place a mark anywhere on the scale below to indicate the value that most reflects the dog's level of itchiness.

| Characteristic | Coat Score Descriptions | | | |
|---|---|---|---|---|
| | 0 | 1 | 2 | 3 |
| Shedding | Minimal amount of shedding.<br><br>Consistently remove less than 10 hairs when gently pulling at fur using thumb and bent index finger on caudal dorsum. | Small amount of shedding.<br><br>Consistently remove from 10-50 hairs when gently pulling at fur using thumb and bent index finger on caudal dorsum. | Moderate amount of shedding.<br><br>Consistently remove hairs too numerous to count when gently pulling at fur using thumb and bent index finger on caudal dorsum. | Severe amount of shedding.<br><br>Consistently remove a clump of hair which is much too many hairs to count when gently pulling at fur using thumb and bent index finger on caudal dorsum. |
| Partial Alopecia | No alopecia. | Small patches of alopecia which do not comprise more than 25 % of the body | Larger patches of alopecia which covers from 25-75 % of the body | Greater than 75 % of the coat is alopecic. |
| Dandruff | No dandruff easily detected when petting fur in a cranial direction starting at the tail head. | Sparse dandruff detected only when petting fur in a cranial direction starting at the tail head. | Dandruff easily detected when petting fur in a cranial direction starting at the tail head. Some may be seen without reverse petting but only on closer examination. | Dandruff easily detected without needing to pet fur in a cranial direction starting at the tail head. |
| Softness | Very Soft | Average softness | Coarse | Coarse and brittle |
| Glossiness | Highly Reflective | Reflective | Minimal reflective quality | Dull and lackluster |
| Greasiness | Dry coat | Normal to minimal amount of greasiness detected with hairs being separate and individual. | Moderate amount of greasiness detected which is starting to cause the fur to gather together. | Severe amount of greasiness detected causing fur to clump together from the grease. |
| Overall Coat Quality for Breed | Above average for breed | Typical coat for breed | Lower than average for breed | Poor coat for breed |

**Figure S3. Skin and Coat Survey Scores and Descriptions.**

**Table S1. Taxa identities (taxa with low identity confidence).**

| Group | Taxa | Identity confidence | BLAST identity |
|---|---|---|---|
| CIHP | *Faecalibaculum* | 0.36 | *Allobaculum* |
| CIHP | *Ligilactobacillus* | 0.61 | *Lactobacillus ruminis* |
| CIHP | *Allobaculum* | 0.50 | *Allobaculum* |
| CIHP | *Turicibacter* | 0.61 | *Turicibacter* |
| CIHP | *Inhella* | 0.41 | *Parasutterella* |
| CIHP | *Romboutsia* | 0.22 | *Clostridium* |

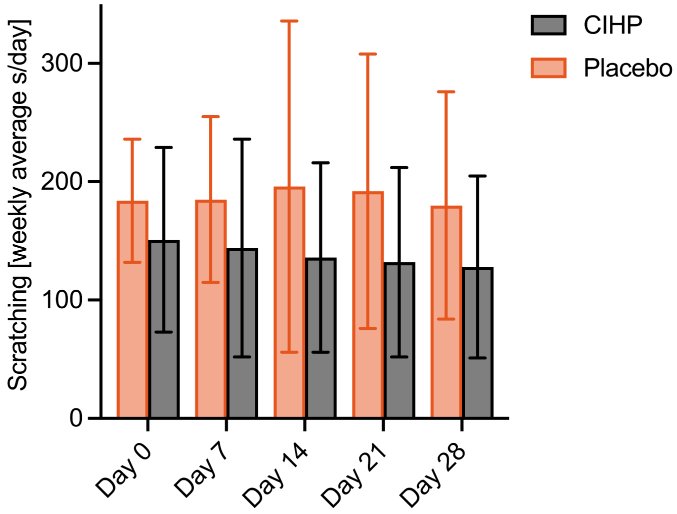


**Figure S4. Daily scratching frequency throughout the study.** Bars indicate the mean and error bars indicate the standard deviation.
